# Current and Future Treatments for Diabetic Retinopathy

**DOI:** 10.3390/pharmaceutics14040812

**Published:** 2022-04-07

**Authors:** Francine Behar-Cohen, Anat Loewenstein

**Affiliations:** 1 Centre de Recherche des Cordeliers, Université de Paris, 75006 Paris, France; 2 Assistance Publique-Hôpitaux de Paris, Department of Ophthalmology, Ophtalmopole, Hôpital Cochin, 75014 Paris, France; 3 Hôpital Foch, 92150 Suresnes, France; 4 Division of Ophthalmology, Tel-Aviv Sourasky Medical Center, Tel Aviv-Yafo 6423906, Israel; anatl@tlvmc.gov.il; 5 Sackler Faculty of Medicine, Tel-Aviv University, Tel Aviv-Yafo 6997801, Israel

The pathogenesis of diabetic retinopathy in humans remains imperfectly understood; in particular, the kinetics of the various pathogenic events in the very early stages of retinal damage are difficult to recognize. Animal models are useful but they do not recapitulate all aspects of human pathology and in particular are not completely transposable to study macular pathology since the majority of laboratory animals do not have a macula.

While for years, diabetic retinopathy has been considered primarily as a microangiopathy, it is becoming increasingly clear that the neurovascular complex and its regulation are affected early in the retina during diabetes and may precede the signs of microangiopathy that are still the first clinically detected signs by simple fundus photography.

However, this microangiopathy would already be a fairly advanced stage in the sequence of biochemical and neuronal deregulations secondary to chronic hyperglycaemia in the retina. The neurovascular complex, which includes not only the vessels and interneurons but also the various glial components of the retina, could be the first site of damage. However, to date, there is no clinically measurable parameter that indicates damage to this neurovascular complex in a meaningful way. The recognized criterion in clinical trials is a gain of three lines of visual acuity, which is not suitable for the evaluation of treatments that would aim to protect the retina from neuronal damage before irreversible complications or macular oedema occur. Neuroprotection is necessary to allow patients to preserve vision in the long term independently of the treatments currently active on macular oedema.

Professor Simo’s review [1] nicely emphasises the molecular targets of the neurovascular complex, particularly those that can be modulated by known drugs that have both vascular and neuronal effects. He also highlights the need to develop a clinical endpoint of its dysfunction, which could be used to develop new treatments.

Several molecules, known for use in other indications, have effects on retinal function in different models of diabetic retinopathy. This is the case of Tauroursodeoxycholic acid (TUDCA), a bile acid, which showed powerful neuroprotective properties in various models of retinopathy [2] and in humans with amyotrophic lateral sclerosis [3].

The group of Jeff Boatright showed that TUDCA starting 1 week after induction of streptozotocin in mice significantly ameliorated retina function evaluated by electroretinography and optokinetic testing [4]. Starting the treatment at 3 weeks was less efficient.

Glibenclamide, well known for its hypoglycaemic properties, has independent neuroprotective properties in the brain and in the retina [5,6,7] as demonstrated by Dr Berdugo-Polak in a type 2 diabetes model. In this paper [6], the long-term oral administration of glibenclamide in Goto kakizaki rats, at non-hypoglycemic doses, was still efficient to reduce signs of retinopathy and the occurrence of retinal edema. Other molecules, recapitulated in Professor Simo’s review [1], such as fenofibrate or calcium dobesilate used in the treatment of dyslipidemia or venous deficiency, respectively, have shown beneficial effects on diabetic retinopathy in large clinical trials, although they are not approved for this indication.

In a more novel way in this indication, a depot formulation of Rho kinase inhibitor is proving interesting to protect the vessels but also the pigment epithelium junctions, with an effect on the extent of ischaemia [8], which few drugs address today.

Dr Lebon, together with Boehringer-Ingelheim’s group, has assessed the slow release of an inhibitor, previously used in humans to prevent stroke complications, and they showed promising results for the clinical use of this compound [8]. Indeed, local intraocular delivery of ROCK inhibitors could restore the ocular barriers and also protect photoreceptor cells [9].

Clinically, anti-VEGF treatments have demonstrated their efficacy in reducing oedema and improving visual function at the cost of repeated injections, as highlighted by Professor Lowenstein in her meaningful review [10]. Intravitreal anti-VEGF agents have indeed significantly improved visual acuity and reduced retinal thickness in patients with diabetic macular edema in long-term follow-up (up to 5 years). They have also provided ≥2-step improvement in the retinopathy severity on color fundus photography in about 30–35% of patients with non-proliferative retinopathy at baseline. Although anti-VEGF agents are the gold standard in the treatment of diabetic macular edema, there is no general consensus regarding their use in patients with various stages of retinopathy.

The other class of drugs used clinically for diabetic macular edema is corticosteroid therapy, the effects of which would be better if dexamethasone implants were used earlier and even as a first-line treatment. However, the risk of intraocular pressure raise and of cataracts still limit their use in young phakic patients.

In the NAVEDEX study, which was a multicenter retrospective observational study, it was demonstrated that good visual gain can still be achieved in patients with low visual receiving the dexamethasone intravitreous implants as first-line treatment [11].

Professor Kodjikian emphasized that Fluocinolone implants have the advantage of lasting several years and are a relevant alternative for diabetics whose retinopathy has been progressing for several years, although the rate of ocular complications is high [12].

In order for new treatments, evaluated in animal models, to find a place in the therapeutic arsenal, it is essential to continue the functional phenotyping of patients at different stages of their disease as recalled in the review of Professor Simo [1]. Recent work has shown that the turnover of micro aneurysms is a predictive marker of complications and visual impairment [13]; thinning of the parafoveal ganglion cell layer occurs before any sign of retinopathy in patients with type 1 diabetes [14]. Other functional tests such as dark adaptation, contrast sensitivity, color vision, and microperimetry showed abnormalities at early stages [15]. Serum biomarkers identified by metabolomics [16], proteomics [17], or hormonal levels [18] could probably also be used, in combination with functional or imaging biomarkers, to predict the diabetic patients more amenable to entering neuroprotection trials. There is no doubt that the development of artificial intelligence will prompt the combination of imaging, functional, and biologic biomarkers as future endpoints for clinical trials.

We thank the authors for the very good reviews and the original work. We also thank the reviewers and the editorial team of Pharmaceutics for their professionalism and efficiency.
